# Why Algae Release Volatile Organic Compounds—The Emission and Roles

**DOI:** 10.3389/fmicb.2019.00491

**Published:** 2019-03-12

**Authors:** Zhaojiang Zuo

**Affiliations:** School of Forestry and Biotechnology, Zhejiang A&F University, Hangzhou, China

**Keywords:** allelopathy, communication, environmental factor, protection, tolerance, volatile organic compounds

## Abstract

A wide spectrum of volatile organic compounds (VOCs) are released from algae in aquatic ecosystems. Environmental factors such as light, temperature, nutrition conditions and abiotic stresses affect their emission. These VOCs can enhance the resistance to abiotic stresses, transfer information between algae, play allelopathic roles, and protect against predators. For homogeneous algae, the VOCs released from algal cells under stress conditions transfer stress information to other cells, and induce the acceptors to make a preparation for the upcoming stresses. For heterogeneous algae and aquatic macrophytes, the VOCs show allelopathic effects on the heterogeneous neighbors, which benefit to the emitter growth and competing for nutrients. In cyanobacterial VOCs, some compounds such as limonene, eucalyptol, β-cyclocitral, α-ionone, β-ionone and geranylacetone have been detected as the allelopathic agents. In addition, VOCs can protect the emitters from predation by predators. It can be speculated that the emission of VOCs is critical for algae coping with the complicated and changeable aquatic ecosystems.

## Introduction

In terrestrial ecosystems, more than 30,000 volatile organic compounds (VOCs) are released from higher plant leaves, flowers and underground parts through secondary metabolism pathway (Peñuelas and Llusià, 2004). These compounds are involved in a broad array of ecological functions and are beneficial to the emitters, such as inhibiting seed germination and seedling growth of other plants (Zuo et al., 2011; Zhang et al., 2012), defensing against herbivores and pathogens (Rapisarda et al., 2012; Bee Park et al., 2013; Zhang et al., 2014), and communicating with other plants (Weir et al., 2004; Baldwin et al., 2006).

**FIGURE 1:**
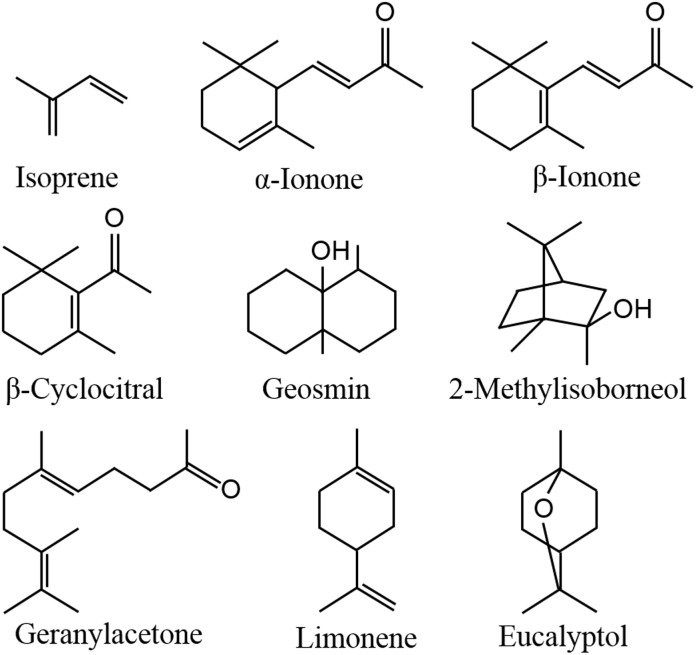
Chemical structures of some main terpenoids in algal VOCs.

In aquatic ecosystems, algae can also release a wide spectrum of VOCs, including terpenoids, furans, sulfo compounds, alkanes, alkenes, alcohols, aldehydes, ketones, and esters (Walsh et al., 1998; Zuo et al., 2012a,b; Xu et al., 2017), which are affected by environmental factors, such as light, temperature, nutrition conditions and abiotic stresses (Bonsang et al., 2010; Zuo et al., 2012a,b; Xu et al., 2017; Ye et al., 2018). Geosmin and 2-methylisoborneol (Figure 1) released from cyanobacteria are two well-known terpenoids (Suurnäkki et al., 2015), as they can cause earthy-musty odor of lake waters (Jüttner, 1995; Huang et al., 2007). Meanwhile, the compounds from the degradation of carotenoids, including β-cyclocitral, β-ionone and geranylacetone (Figure 1), also contribute to the water odor (Jüttner, 1979, 1984). It is not clear whether these odor compounds influence human health, but they dramatically impact water supplies by decreasing esthetic quality and increasing the costs of water treatment. In addition to causing water odor, algal VOCs serve important functions in enhancing emitters’ tolerance, communicating with homogeneous algae, playing allelopathic roles in heterogeneous algae and aquatic macrophytes, and protecting against predators, which may be the true reason for algae releasing VOCs, benefiting to the survival and propagation of emitters. In this review, the emission and roles of algal VOCs are summarized.

## Environmental Factors Affecting VOC Emission From Algae

### Light

**FIGURE 2:**
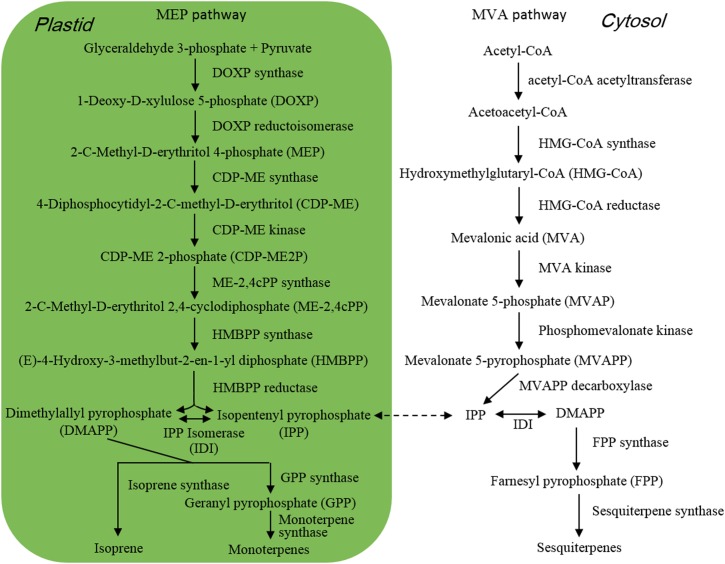
Pathway of terpene synthesis.

Isoprene (Figure 1) is composed of 5 carbon atoms and called hemiterpene, the minimum terpenoids. Cyanobacteria, diatoms and green algae can release it with dependence on light condition (Shaw et al., 2003), e.g., the isoprene emission rate from *Prochlorococcus* increased with raising light intensity (Bonsang et al., 2010). When *Thalassiosira weissflogii*, *T. pseudonana*, *Pleurochrysis carterae*, and *Rhodomonas salina* were kept in different light intensity for 4 h, isoprene was the maximum released compound with some monoterpenes which are composed of the isoprene C5 unit, and high light intensity showed promoting effect on isoprene emission (Meskhidze et al., 2015). Isoprene and monoterpenes are synthesized in plastids via methylerythritol-4-phosphate pathway (MEP) (Figure 2; Rohmer et al., 1993), and are released from algae after direct synthesis, due to no storage structure. Light promotes their emission, as the availability of energetic cofactors and C intermediates increases the availability of dimethylallyl pyrophosphate (DMAPP), immediate precursor of isoprene and monoterpenes in MEP (Rasulov et al., 2009; Niinemets and Sun, 2015).

In marine algae, halogenated hydrocarbons are common compounds in their VOCs. *Solieria chordalis* released 9 halogenated hydrocarbons, such as CH_3_I, CH_3_CH_2_I, CH_2_ClI, CH_2_Br_2_, CHBrCl_2_, CHBr_2_Cl, CH_2_BrI, CHBr_3_ and CH_2_I_2_, of which emission rate increased in the light but declined in the dark (Bondu et al., 2008). During a day, the highest production rate of halogenated hydrocarbons from *Hypnea spinella* and *Falkenbergia hillebrandii* was observed at mid-day (Ekdahl et al., 1998). The formation of halogenated hydrocarbons depends on the haloperoxidases that catalyze H_2_O_2_ oxidizing halide ions to form halogenated compounds (Ohsawa et al., 2001; Winter and Moore, 2009). H_2_O_2_ can be directly produced and indirectly transformed from other reactive oxygen species (ROS) in cells (Milne et al., 2009). High light intensity leads to massive ROS production and then promotes the formation and emission of halogenated hydrocarbons (Hughes and Sun, 2016).

### Temperature

When *Pleurosira laevis* and *Enteromorpha flexuosa* were kept at 17°C and 23°C, higher temperature promoted the emission of CHCl_3_ from *P. laevis* and CHBr_3_ from *E. flexuosa* (Abrahamsson et al., 2003). Heat shock increased the emission of C6 green leaf volatiles (GLVs) and carotenoid degradants from *Lobaria pulmonaria*, a symbiont of fungus, cyanobacterium *Nostoc* and green alga *Dyctiochloropsis* (García-Plazaola et al., 2017). GLVs mainly include C6 alcohols and aldehydes, which are formed via oxidative degradation of fatty acids (Peñuelas and Llusià, 2004). β-Cyclocitral, α-ionone (Figure 1), β-ionone and geranylacetone are typical carotenoid degradants in cyanobacteria (Jüttner, 1979, 1984; Ikawa et al., 2001). High temperature can induce the production of massive ROS in algae, which benefits to the oxidation of halide ions, fatty acids and carotenoids, leading to the formation of halogenated hydrocarbons, GLVs and carotenoid degradants.

### Nutrition Conditions

In water bodies, the multiple nutrition conditions, mainly phosphorus (P) and nitrogen (N) forms and levels, can influence the emission of VOCs from algae. Polyphosphate (PolyP) and orthophosphate widely exist in water bodies (Nishikawa et al., 2006). When two typical algal species of cyanobacterial bloom *Microcystis flos-aquae* and *M. aeruginosa* were kept in the medium with K_2_HPO_4_, sodium pyrophosphate and sodium hexametaphosphate as the sole P source, they released different amount and components of VOCs, mainly including furans, sulfo compounds, terpenoids, benzenoids, hydrocarbons, aldehydes and esters. Meanwhile, non-P condition showed the maximum promoting effect on the VOC emission (Ye et al., 2018; Zuo et al., 2018b). In the field works, a negative relationship between geosmin amount and P concentration in reservoirs has also been found (Dzialowski et al., 2009). In aquatic ecosystem, P is considered as a limiting nutrient for algal massive growth, due to its easy precipitation as insoluble salts (Qian et al., 2011; Tekile et al., 2015). Under that condition, algae released maximum VOCs, which were beneficial to the emitters competing nutrients by inhibiting other algae (Yang et al., 2018; Zuo et al., 2018b).

When *M. flos-aquae* and *M. aeruginosa* were kept in different N forms such as NaNO_3_, NaNO_2_, NH_4_Cl, urea, serine, lysine, and arginine, they released different amount and components of VOCs, and the emission amount increased with reducing N concentration, with the maximum emission under non-N condition (Xu et al., 2017; Zuo et al., 2018a). Similarly increased emission of alcohols and β-cyclocitral was also detected when *M. aeruginosa* cells exhausted nitrate N nutrient after 35 days (Hasegawa et al., 2012). Under non-N condition, *M. aeruginosa* cells significantly up-regulated the expression of 4 genes which encoded pyruvate kinase, malic enzyme, phosphotransacetylase and aspartate aminotransferase, respectively (Zuo et al., 2018a). Pyruvate and acetyl-CoA are immediate precursors of isoprene and monoterpenes in MEP and sesquiterpenes in mevalonate pathway (MVA) (Figure 2), respectively. Pyruvate kinase catalyzes the formation of pyruvate by transferring a phosphate group from phosphoenolpyruvate to adenosine diphosphate (ADP) (Zhang et al., 2017). Malic enzyme and phosphotransacetylase are involved in the formation of acetyl-CoA (Kyrtopoulos and Satchell, 1972; Ikaran et al., 2015). Benzenoids and phenylpropanoids are considered as the second largest class of VOCs after terpenoids (Dudareva et al., 2013; Sun et al., 2016), which are mainly derived from phenylalanine. Aspartate aminotransferase functioned in the synthesis of phenylalanine in the last step of shikimate pathway (Maeda et al., 2011). Non-N condition induced the expression of genes that were involved in the formation of terpenoid and benzenoid precursors and promoted VOC emission (Zuo et al., 2018a).

### Abiotic Stresses

*Chlamydomonas reinhardtii* is a model material for algal research, and released plenty of VOCs, including alkanes, alkenes, terpenoids, alcohols, aldehydes, ketones, and esters. Their emission amount and components increased when the cells were stressed by acetic acid, NaCl and Na_2_CO_3_ (Zuo et al., 2012a,b, 2015). Meanwhile, GLVs were induced to release under acetic acid and NaCl stresses, but not under Na_2_CO_3_ stress (Zuo et al., 2012a,b). Similarly, NaNO_3_ stress promoted the emission of terpenoids, sulfocompounds, benzenoids, aldehydes and esters from *M. flos-aquae* and *M. aeruginosa* cells (Gan et al., 2015). Under salt stress, *Solieria chordalis* and *Gymnogongrus antarcticus* increased the emission of halogenated hydrocarbons, which may result from the increased ROS under the stress (Laturnus et al., 2000; Bondu et al., 2008).

Although aquatic environment is relative stable compared to terrestrial environment, algae are easily influenced by environmental factors, due to their difficult movement and fast migration by water flow. They adjust VOC synthesis and increase the emission under abiotic stresses, such as high light, warmer temperature, nutrient deficiency, increased salinity and acidity. These VOCs perform important ecological functions (Watson, 2003; Fink, 2007; Zuo et al., 2012a, 2018b), which should be survival strategies for the emitters and their population.

## Ecological Functions of Algal VOCs

### Lowering Oxidative Stress in Algae

Abiotic stresses induce the production of massive ROS in algal cells, indicating that algae not only suffer the direct abiotic stresses but also the indirect oxidative stress. Although ROS are important signaling molecule, their massive accumulation can damage the photosynthetic apparatus, cell membranes, proteins and DNA (Affenzeller et al., 2009; Tartoura and Youssef, 2011; Zuo et al., 2014; He et al., 2015), and even induce programmed cell death (PCD) (Affenzeller et al., 2009; Chen et al., 2019). In higher plants, their VOCs especially isoprene and monoterpenes have been recognized as the antioxidant agents to scavenge ROS and protect cell membrane and photosynthetic apparatus under several abiotic stresses, such as high temperature, drought, salinity, ozone, etc. (Vickers et al., 2009; Schaub et al., 2010; Velikova et al., 2011; Zuo et al., 2017). Isoprene and monoterpenes were released from several algae under abiotic stresses (Zuo et al., 2012a,b; Meskhidze et al., 2015; Xu et al., 2017), indicating that they might play antioxidative roles under stresses. pH 5.0 acetic acid induced *C. reinhardtii* cells to undergo PCD, and O_2^-^_ and H_2_O_2_ rapidly accumulated to high levels in the cells at the beginning of the PCD and reduced during the process. During the ROS decrease, antioxidant enzymes did not contribute too much in scavenging ROS, as their activities declined quickly and even disappeared. However, VOCs may play important roles in scavenging or adjusting ROS levels, due to the dramatic increase of oxygenated compounds, including ketones, esters and aldehydes (Zuo et al., 2015). In algae, the massively produced ROS may be used to oxidize halide ions, fatty acids and carotenoids to lower oxidative damage, with emission of halogenated hydrocarbons, GLVs and carotenoid degradants (Jüttner, 1984; Ohsawa et al., 2001; Peñuelas and Llusià, 2004; Winter and Moore, 2009). It can be speculated that the production and emission of VOCs are beneficial to algal cells resisting ROS under abiotic stresses.

### Inducing Defense in Homogeneous Algae

When healthy *C. reinhardtii* cells were exposed to the VOCs from *C. reinhardtii* undergoing PCD, their normal growth declined but the activities of antioxidant enzymes increased (Zuo et al., 2012a). Similar effects were also found when healthy *C. reinhardtii* cells were exposed to the VOCs from the cells under NaCl and NaCO_3_ stresses (Zuo et al., 2012b). *C. reinhardtii* released NO and ethylene during PCD induced by wasp venom, and the two volatile molecules were considered as the information compounds that transferred information for other healthy *C. reinhardtii* cells, as the healthy cells treated with the solution that underwent PCD were not induced PCD by wasp venom (Yordanova et al., 2010). These results indicate that stress-induced VOCs are information agents that transfer message to other homogeneous cells and induce them to make a preparation for the upcoming stresses. This message transfer has been well studied between higher plants, which is called “cross-talk trees” and may derive from the primitive organism algae (Weir et al., 2004; Baldwin et al., 2006; Kessler et al., 2006). The information transfer mechanism serves the same function in algae and plants, which can avoid them from the sustaining defense consumption and be beneficial to the growth and propagation of the population.

### Allelopathic Effects on Other Heterogeneous Algae and Aquatic Macrophytes

In eutrophicated water, cyanobacteria massively grow and dominate the water bodies with reduction and even disappearance of other algae and aquatic macrophytes. It is well known that algal toxins from cyanobacteria play an important allelopathic role during the process (Suikkanen et al., 2004; Fink, 2007; Li and Li, 2012). Similarly, the VOCs from cyanobacteria have also been found as the allelopathic agents for other algae (Watson, 2003). When *Chlorella vulgaris* was exposed to the VOCs from *M. flos-aquae* under non-N condition, remarkable decreases were detected in the cell growth, photosynthetic pigment content and photosynthetic abilities (Xu et al., 2017). Similar results were also found in *C. reinhardtii* cells in exposure of the VOCs from *M. flos-aquae* and *M. aeruginosa* under non-P condition (Yang et al., 2018; Zuo et al., 2018b). For aquatic macrophytes, there are very limited reports about their reduction which results from cyanobacterial VOC allelopathy. When *Lactuca sativa* seeds were exposed to dimethyl disulfide, their germination was inhibited markedly (Gómez-Tenorio et al., 2015). In addition, *C. vulgaris* VOCs showed inhibitory effects on the α-amylase activity and coleoptile growth of barley (Abdel-Baky et al., 2002), indicating that algal VOCs might also have allelopathic effects on macrophytes. In eutrophicated water, the massive growth of cyanobacteria will compete with nutrients with other algae and aquatic macrophytes, which easily result in the lack of nutrients, especially N and P. This lack can induce abundant VOC emissions from cyanobacteria to keep the emitters’ competitive advantage for nutrients by inhibiting other competitors.

Among the abundance of VOCs from *Microcystis*, limonene and eucalyptol (Figure 1) showed inhibitory effects on *C. vulgaris* and *C. reinhardtii* cell growth by inducing photosynthetic pigment degradation and declining photosynthetic abilities (Zhao et al., 2016; Zhou et al., 2016). β-Cyclocitral, α-ionone, β-ionone and geranylacetone were also the main compounds in *Microcystis* VOCs, which showed inhibitory effects on *C. pyrenoidosa* cell growth (Ikawa et al., 2001). Meanwhile, β-cyclocitral of 0.1–0.5 mg⋅ml^-1^ can cause cell rupture of *Nitzschia palea* (Chang et al., 2011). Moreover, high concentration β-cyclocitral even impact the growth of *Microcystis*, by causing lysis (Ozaki et al., 2008). These results suggest that these compounds may be the main allelopathic agents in cyanobacterial VOCs, but the agents with high concentration may be detrimental to the emitters.

### Protecting Algae Against Predators

Algae are the primary producers in aquatic ecosystems, which are preyed by predators as food. VOCs play important roles in protecting algae from predation (Fink, 2007). When diatom cells were damaged, they released polyunsaturated eicosapentaenoic acid which was toxic for crustacean herbivores (Jüttner, 2001). Meanwhile, diatoms can release polyunsaturated aldehydes to repel herbivorous zooplankton (Jüttner, 2005) and inhibit sea urchin laying eggs (Miralto et al., 1999). After damage, *Thalassiosira rotula* released 2,4-decadienal and 2,4,7-decatrienal which were converted from free fatty acids by lipoxygenases to defense (Pohnert, 2002). Compared to *Dictyopteris membranacea* with releasing C11 sulfocompounds, *Ampithoe longimana* tended to feed *Dictyopteris hoytii* without releasing the compounds (Schnitzler et al., 2001). In the treatments with β-cyclocitral and 2,4,7-decatrienal, the swimming velocity of *Daphnia magna* increased significantly (Watson et al., 2007; Jüttner et al., 2010), indicating that the two compounds can protect algal cells by repelling the predators. Meanwhile, polyunsaturated fatty acids showed inhibitory effects on *D. magna* laying eggs (Martin-Creuzburg and von Elert, 2009). Although sterols produced from algae were not detected in the VOCs, they impacted the growth and reproduction of *Daphnia* (Martin-Creuzburg and von Elert, 2004; Martin-Creuzburg et al., 2014) and sexual development of sea scallop (Wang and Croll, 2004) due to their roles as hormones, indicating these compounds should also be protective agents. These protective mechanisms are also reserved in higher plants to repel insects or impact their development with more compounds (Rapisarda et al., 2012; Bee Park et al., 2013; Zhang et al., 2014), which are crucial to algae and plants protecting themselves against predators.

## Prospection

In aquatic ecosystems, algae release an abundance of VOCs to increase their tolerance to abiotic stresses, transfer stress information to homogeneous algae to induce defense, play allelopathic roles on heterogeneous algae and aquatic macrophytes for competing nutrients, or protect against predators. The functions of VOCs in algae are very similar to that in higher plants. However, the studies about algal VOCs, especially their roles, are still in the primary stage. For emitters, how do they release the VOCs to response different environmental conditions? For acceptors, it is important for them to identify and sense the signaling molecule and make a correct response further. Algal VOCs are a blend of compounds. Which are the exact information agents among them? What is the exact information for each agent? These questions should be answered in future studies to uncover the VOC communication by using molecular biology and technology. Undoubtedly, the emission of VOCs from algae is critical and beneficial to the survival and reproduction of emitters and their population in response to the complicated and changeable aquatic ecosystems.

## Author Contributions

The author confirms being the sole contributor of this work and has approved it for publication.

## Conflict of Interest Statement

The author declares that the research was conducted in the absence of any commercial or financial relationships that could be construed as a potential conflict of interest.
